# Supplementary material for: Association between oral microbiome and seven types of cancers in East Asian population: a two-sample Mendelian randomization analysis
Source: Front Mol Biosci. 2023 Nov 21;10:1327893. doi: 10.3389/fmolb.2023.1327893 (PMC10702768; doi:10.3389/fmolb.2023.1327893)
Supplement: Supplementary file 1 [file DataSheet1.ZIP › Supplementary Materials/MR plots for tongue/tongue═╝/Pancreatic cancer/╥╚╧┘░⌐_╔╕╤í╜ß╣√/pheno.3515_to_pancreatic cancer_forest.pdf]

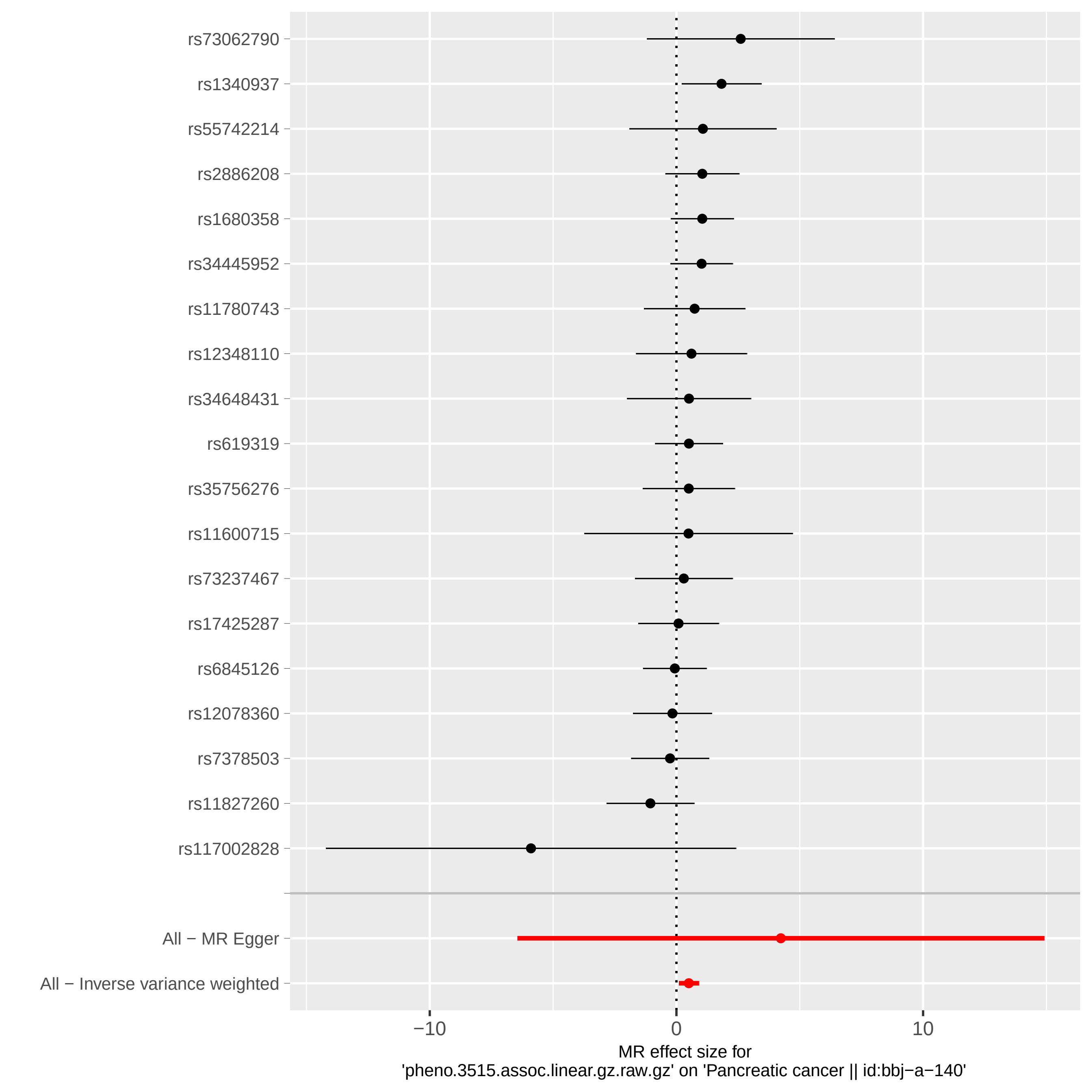
rs73062790
rs1340937
rs55742214
rs2886208
rs1680358
rs34445952
rs11780743
rs12348110
rs34648431
rs619319
rs35756276
rs11600715
rs73237467
rs17425287
rs6845126
rs12078360
rs7378503
rs11827260
rs117002828
All − MR Egger
All − Inverse variance weighted
−10
0
10
MR effect size for
'pheno.3515.assoc.linear.gz.raw.gz' on 'Pancreatic cancer || id:bbj−a−140'
